# Crushed White Wheat

**Published:** 1874-11

**Authors:** 


### CRUSHED WHITE WHEAT.

We question if there is a better food than this article, manufactured by F. E. Smith & Co., at the Atlantic Flour Mills, Hamilton Avenue, Brooklyn. While the appetite not unfrequently inclines to reject the commoner kinds of "cracked wheat," it speedily comes to crave this delicate, sweet and light-colored crushed grain. We are constantly using it in our family, and can say, truthfully, that our "children cry for it." Eaten with cream and sugar it is a delicious dish. With this bruised grain as a basis, a multitude of most appetising and healthful dishes can be fabricated, as may be seen by a pamphlet issued by Messrs. Smith & Co., which they will send on application.

